# Appointments

**Published:** 1853-09

**Authors:** 


*Appointments.*—Dr. Socrates Maupin has been appointed to the chair of Chemistry, in the University of Virginia, vacated by the resignation of Dr. J. Lawrence Smith.

The following new appointments have been made in the Medical College of Ohio: Dr. G. W. Bayless, Anatomy; Dr. Asbury Evans, Surgery; Dr. Charles W. Wright, Chemistry; Dr. N. T. Marshall, Midwifery; Dr. Samuel G. Armour, Physiology and Pathology.

Dr. E. H. Parker, editor of the New Hampshire Journal of Medicine, has been appointed Prof. of Physiology and Pathology, in the New York Medical College.

